# Dentine

**Published:** 1869-05

**Authors:** Henry S. Chase


### ARTICLE IX.

### *Dentine.*

By Prof. HENRY S. CHASE, M. D.

Dentine is that anatomical element of which the great mass of a tooth is composed. It gives shape to the crown and the roots In the crown it is covered by the enamel, which extends as far towards the roots as the neck of the tooth, a little below the margin of the gums. In the roots, it is covered on the outside by the cementum, which latter is composed wholly of osteal cells. The nerve or pulp canal, which passes through the roots, is lined also with cementum for a short distance from the foramini of the roots, towards the crown ; thus covering the internal surfaces of the dentine in the roots with osteal cells.

Dentine is composed of tubes opening on the interior of the tooth, or that chamber which is called the pulp cavity, or nerve cavity, and which contains the pulp of the tooth. The direction of these tubuli is from the pulp cavity towards the exterior of the tooth, radiating and giving off smaller tubules as it passes along, like the branches of an artery. Some tubes give off no branches, but run in an undulating manner, without branching, until they terminate at the base of the enamel.

The dentine tubes in the roots make connection with the canaliculi of the cementum cells. The tubules carry the colorless blood to every portion of the tooth, thus giving it nourishment. There are no vessels carrying red blood into the dentine, or capable of doing so, even in inflammation, for the diameter of their interior is not more than the ten thousandth part of an inch. The dark appearance sometimes observed in teeth is owing to disintegration of the blood discs, which may then permeate the dental tubule.

The blood which the latter receive comes from the vessels of the dental pulp, principally, although some is received from the cementum cells, which lie in close connection with the pericementum or dental periosteum.

Besides conveying nourishment to the enamel and intertubular dentine, the tubes contain nerve fibrils, which are received from the dental pulp, and these nerve fibrils run the whole length of the tubes.

It has been observed that the tubes penetrate the enamel in some instances, running between its roots nearly to the surface. This fact accounts for the pain often felt in a tooth unaffected by decay, from the contact of acids, of sweets, and from the pressure between the teeth of woolen cloth and some other substances.

The microscope has not actually demonstrated that the fibrils are nerves. But it has been demostrated that every dental tubule contains a soft fibril which may be drawn out of the tubes to a considerable extent before being broken off. Facts in dental physiology and pathology make it almost certain that these fibrils are true nerves.

There is an inter tubular substance in dentine which is wholly composed of lime salts. These territories are without sensation. There are no fibrils there. In dental decay we find portions of the cavity very sensitive to the touch of an instrument, and other parts totally without sensibility.

The history of dentine shows that a tooth is not dead when the pulp is dead and removed from the tooth, as we have seen that the dental tubes anastomoze with the canaliculi of the cementum; and the cementum cells are nourished from the periosteum of the roots with which they are united. Observation has taught us that a tooth may be sensitive after the removal of its nervous pulp, the dentine, within a cavity of decay, proving extremely sensitive to the touch of an excavator in some instances, thus showing the presence of nerve fibrils, received from the pericementum or dental periosteum.—*Medical Investigator.*

---

